# Changes in the Concentrations of Proangiogenic Cytokines in Human Brain Glioma and Acute Lymphoblastic Leukemia

**DOI:** 10.3390/ijms25052586

**Published:** 2024-02-23

**Authors:** Lukasz Oldak, Zuzanna Zielinska, Patrycja Milewska, Sylwia Chludzinska-Kasperuk, Eryk Latoch, Katarzyna Konończuk, Maryna Krawczuk-Rybak, Aleksandra Starosz, Kamil Grubczak, Joanna Reszeć, Ewa Gorodkiewicz

**Affiliations:** 1 Bioanalysis Laboratory, Faculty of Chemistry, University of Bialystok, Ciolkowskiego 1K, 15-245 Bialystok, Poland; 2 Doctoral School of Exact and Natural Science, Faculty of Chemistry, University of Bialystok, Ciolkowskiego 1K, 15-245 Bialystok, Poland; 3 Biobank, Medical University of Bialystok, Waszyngtona 13, 15-269 Bialystok, Poland; 4 Department of Pediatric Oncology and Hematology, Medical University of Bialystok, 15-274 Bialystok, Poland; eryklatoch@gmail.com (E.L.);; 5 Department of Regenerative Medicine and Immune Regulation, Medical University of Bialystok, Waszyngtona 13, 15-269 Bialystok, Poland; aleksandra.starosz@umb.edu.pl (A.S.);; 6 Department of Medical Pathology, Medical University of Bialystok, Waszyngtona 13, 15-269 Bialystok, Poland

**Keywords:** VEGF-A, VEGF-R2, FGF-2, glioma, childhood acute lymphoblastic leukemia, biomarkers

## Abstract

Acute lymphoblastic leukemia (ALL) and glioma are some of the most common malignancies, with ALL most often affecting children and glioma affecting adult men. Proangiogenic cytokines and growth factors play an important role in the development of both of these tumors. Glioma is characterized by an extremely extensive network of blood vessels, which continues to expand mainly in the process of neoangiogenesis, the direct inducers of which are cytokines from the family of vascular endothelial growth factors, i.e., vascular endothelial growth factor (VEGF-A) and its receptor vascular endothelial growth factor receptor 2 (VEGF-R2), as well as a cytokine from the fibroblast growth factor family, fibroblast growth factor 2 (FGF-2 or bFGF). Growth factors are known primarily for their involvement in the progression and development of solid tumors, but there is evidence that local bone marrow angiogenesis and increased blood vessel density are also present in hematological malignancies, including leukemias. The aim of this study was to examine changes in the concentrations of VEGF-A, VEGF-R2, and FGF-2 (with a molecular weight of 17 kDa) in a group of patients divided into specific grades of malignancy (glioma) and a control group; changes of VEGF-A and FGF-2 concentrations in childhood acute lymphoblastic leukemia and a control group; and to determine correlations between the individual proteins as well as the influence of the patient’s age, diet, and other conditions that may place the patient in the risk group. During the statistical analysis, significant differences in concentrations were found between the patient and control groups in samples from people with diagnosed glioma and from children with acute lymphoblastic leukemia, but in general, there are no significant differences in the concentrations of VEGF-A, VEGF-R2, and FGF-2 between different grades of glioma malignancy. Among individuals treated for glioma, there was no significant impact from the patient’s gender and age, consumption of food from plastic packaging, frequency of eating vegetables and fruit, smoking of tobacco products, the intensity of physical exercise, or the general condition of the body (Karnofsky score) on the concentrations of the determined cytokines and receptor. The listed factors do not bring about an actual increase in the risk of developing brain glioma.

## 1. Introduction

Acute lymphoblastic leukemia (ALL) is a genetically heterogeneous lymphoid neoplasm. It originates from progenitor cells of B and T lymphocytes. Differences in morphological, immunophenotypic, and genetic features between normal progenitor cells and those affected by the disease allow the diagnosis of hematopoietic and non-hematopoietic cancers [1]. ALL is the most common malignancy in children. Advances in diagnosis and therapeutic methods have resulted in an 80% 5-year relapse-free survival rate and a 90% 8-year survival rate [2], and the latest data even report a 5-year survival rate of 93.5%, using the most effective chemotherapy methods [3]. Unfortunately, in developing countries, morbidity rates are high and survival rates are poor. This state of affairs is influenced by several conditions, including the low level of public awareness of the disease, the use of pesticides without proper marking of the area and protection of people, and the lack of access to modern medical care [3].

Glioma is one of the most common primary tumors of the central nervous system in adults. This and other cancers of the central nervous system together account for approximately 3% of all cancers worldwide, with men at higher risk. Survival prognosis varies depending on the patient’s age and histological type of cancer, but in general, once diagnosed and treated, a patient does not live longer than five years. The prognosis is particularly poor for glioblastoma multiforme and in the elderly. In highly developed countries, an increase in recorded cases and, at the same time, an increase in the incidence of disease has been observed. This situation is largely caused by the increase in public awareness and improvement of medical care and the availability of modern therapies and diagnostic methods [4].

A characteristic feature of glioma is the presence of an extensive network of blood vessels. The expansion of the tumor and its growth, therefore, depend on its ability to produce its own vascularization. Each new cancerous tumor needs an extremely extensive network of blood vessels. Otherwise, no tumor can grow larger than about 2 mm [5]. Several factors influence the formation of new blood vessels, including vascular endothelial growth factor (VEGF-A) and its receptor 2 (VEGF-R2), and basic fibroblast growth factor (bFGF, FGF-2) [5,6].

A biological feature of all diffuse gliomas is the infiltration of cancer cells. They have the ability to move long distances from their place of origin, and this ability is provided by tracking neuropilin structures. Cancer cells can even move to the opposite hemisphere and cause further cancer foci, which leads to what is called multifocal glioma. Due to all of the factors mentioned, complete microscopic resection of the tumor can never be achieved. Additionally, remaining cancer cells contribute to disease recurrence [7].

In 2021, updated guidelines for the diagnosis and treatment of adults with disseminated gliomas, proposed by the European Association of Neuro-Oncology (EANO), were published. The section on diagnostics is based on the update of the classification of tumors of the central nervous system proposed by WHO in 2016 and the recommendations of the “Consortium to Inform Molecular and Practical Approaches to CNS Tumor Taxonomy—Not Officially WHO (cIMPACT-NOW)”.

Recommendations regarding therapy were formulated based on the results of the latest clinical trials [8]. The fifth edition of the WHO Classification of Tumors of the Central Nervous System (WHO CNS5) includes significant changes to enhance the role of molecular diagnostics in the classification of CNS tumors, while maintaining other well-established approaches to tumor characterization, including histology and immunohistochemistry. So far, the classification of CNS tumors has been based on the results of histological tests, supported by auxiliary tests such as immunohistochemistry. However, the development of molecular markers has contributed to providing both auxiliary and defining diagnostic information, and therefore, classification based only on histological tests has been abandoned. The latest classification broadly divides gliomas into limited and diffuse. The new approach to the classification of gliomas, neuroglial tumors, and neuronal tumors, which was adopted in WHO CNS5, uses a division into six different families, presented in Table 1.

Another category is choroid plexus tumors with distinct epithelial features, which have been treated separately from the set of six families listed above [9].

The current classification of gliomas according to WHO guidelines (WHO CNS5), taking into account the grades of glioma malignancy, is presented in Table 2 [10,11,12,13].

**Figure 1:**
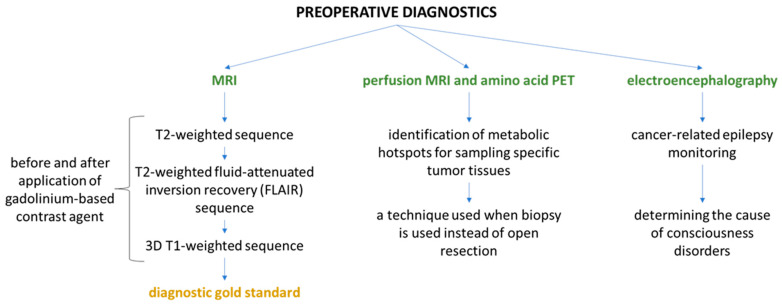
Scheme of preoperative diagnosis of brain glioma.

Preoperative diagnosis is based on imaging methods and examination of bioelectric brain activity. The characteristics and application of each method are presented in the diagram below (Figure 1) [14,15,16].

Glioma cells, particularly glioblastoma multiforme, are a rich source of angiogenic factors, especially VEGF-A. VEGF-A signaling in mammals is a very complex process that also involves molecules of the appropriate receptor. VEGF-R2 has been considered to be the main mediator of VEGF-A bioactivity. Specific biological responses of the VEGF-A and VEGF-R2 systems mediate both physiological and pathological angiogenesis. Signaling pathways are very complex and not yet fully understood. Their complexity is additionally increased by the interactions of VEGF-R2 with coreceptors, which include, among others, neuropilins, heparan sulfate proteoglycans, and αvβ3 integrin [6]. VEGF secreted from primary tumors promotes tumor progression and induces angiogenesis through VEGF-R receptors on endothelial cells. It also has the ability to send signals directly through its receptors, which are present in cells of hematopoietic origin and in various cancer cells. When VEGF binds to VEGF-R, dimerization and oligomerization occur, which activate the tyrosine kinase activity of VEGF-R. Activation of tyrosine kinase involves autophosphorylation and transphosphorylation of tyrosine residues in the cytoplasmic domain. Overexpression of VEGF and activation of VEGF-R contribute to an increase in cancer cell motility [17]. Another potent pro-angiogenic factor is basic fibroblast growth factor (FGF-2 or bFGF). It has the ability to increase proliferation and migration and control the survival of endothelial cells, and it also directly affects the angiogenesis process. Although the mechanism of action of VEGF and FGF-2 differs, there is a functional relationship between these factors. VEGF overexpression is able to increase FGF-2 expression and vice versa. Both of these factors work synergistically. Moreover, both factors are so interdependent that overexpression of FGF-2 may be responsible for increasing the resistance of the tumor to therapy directed against VEGF-A. However, simultaneous inhibition of VEGF-A and FGF-2 signaling results in the inhibition of tumor growth [18,19].

Angiogenesis plays a fundamental role primarily in the growth of solid tumors, but it is also essential during the pathogenesis of hematological malignancies [20,21]. Studies indicate that local bone marrow angiogenesis combined with increased blood vessel density is important for both the development of acute myeloid leukemia and sensitivity to chemotherapy. There is also evidence that increased microcirculation occurs in patients with leukemia as well as those with lymphoma. They have also been found to have increased levels of pro-angiogenic factors, mainly VEGF-A. It has also been shown that increased levels of receptors, including VEGF-R2, predict worse survival rates for patients with acute lymphoblastic leukemia (ALL); therefore, growth factors and their receptors are actively involved in the pathogenesis of hematological cancers in both children and adults [22]. 

FGF-2 is associated with the progression and development of hematological cancers. Elevated levels of FGF-2 immunoreactivity have been reported in patients with hematologic diseases such as chronic myeloid and lymphoid leukemia, hairy cell leukemia, and non-Hodgkin’s lymphoma. Moreover, increased FGF-2 immunoreactivity significantly correlates with an increased risk of chronic lymphocytic leukemia (CLL) and, at the same time, with a reduced susceptibility to apoptosis. Moreover, FGF-2 may have a protective effect against apoptosis in lymphocytes in CLL patients. In addition, FGF-2 released by leukemic cells may support tumor progression by directly stimulating bone marrow angiogenesis [23].

In addition to the previously mentioned proteins that have a strong connection with angiogenesis, others also act as activators of angiogenesis. These include hepatocyte growth factor (HGF), angiopoietin-1 (ANG-1), and angiopoietin-2 (ANG-2), but in the case of the latter two, the angiogenic process is more complex. ANG-1 and ANG-2 bind to their receptor TIE-2, but TIE-2 signaling can promote or inhibit angiogenesis, among others, by influencing endothelial cell survival, vessel growth, or the presence of other angiogenic factors [24]. Pro-angiogenic factors are also an important therapeutic target. Antiangiogenic drugs exert their therapeutic effects by blocking specific receptors, but none of them can completely block all receptors and prevent further signaling. Antiangiogenic drugs are most often monoclonal antibodies, sold under various trade names, e.g., Bevacizumab, and synthetic inhibitors, e.g., Sunitinib, Sorafenib, Pazopanib [25]. Increased angiogenesis in lung tissue has also been proven, which is associated with the proliferation of endothelial cells, which in turn are responsible for the development of pulmonary hypertension. Moreover, chronic obstructive pulmonary disease (COPD) is associated with significantly reduced capillary density, leading to a state of hypoxia, which in turn activates hypoxia-inducible factor (HIF-1α), also important for angiogenesis. In turn, asthma is characterized by airway congestion and remodulation caused by inflammation and mucus plug deposition in the airways, and these factors are also associated with angiogenesis [26]. 

Surface plasmon resonance imaging (SPRi) is an imaging technique for visualizing changes in refractive index. It is based on an oscillation of charge density occurring at the junction of two media characterized by dielectric constants of opposite signs [27]. It enables the observation of interactions between biomolecules at the nanoscale. This technique has attracted the interest of researchers due to its high sensitivity, the absence of the need to use dedicated labeled antibodies, the simplicity of the equipment, quick detection, and high throughput [28,29]. Over recent years, the evolution of the SPR technique has enabled its use in clinical diagnostics, environmental monitoring, and drug research. In addition to the kinetic and thermodynamic characteristics of biological systems, SPR (including SPRi) enables high-throughput, and therefore, fast and low-cost, quantitative analysis of biomarkers in body fluids, without the need for special preparation of the sample for measurement [30,31]. 

In this study, we present the results of determinations of VEGF-A, VEGF-R2, and FGF-2 in the plasma of patients with cerebral glioma in all four grades and in a control group, and results of quantitative analysis of VEGF-A and FGF-2 in the serum of children with acute lymphoblastic leukemia and in a control group. The conducted research was aimed at examining the relationship between changes in the concentration of pro-angiogenic cytokines between the group of patients and the control group in glioma and acute lymphoblastic leukemia, determining the strength and direction of interactions between the determined cytokines depending on the disease and determining the diagnostic usefulness of the VEGF-A, VEGF-R2, and FGF-2 determination methods for glioma and VEGF-A and FGF-2 for leukemia.

We examined the influence of glioma grade on all markers determined simultaneously, and the influence of the molecular markers IDH 1/2, p53, and EGFR. We also examined the correlation between the concentrations of the determined biomarkers and parameters available in the clinical description using a multiple regression model (in the case of samples from glioma patients). Due to the inability to obtain all of the necessary clinical data on samples from children with ALL, we compared the results of the quantitative analysis of VEGF-A and FGF-2 only with the concentrations of the same biomarkers in the serum of children without diagnosed cancer or inflammatory lesions. Nevertheless, the research may provide further evidence concerning the involvement of growth factors in hematological cancers.

## 2. Results

The analysis began with the examination of the normality of the distribution of the data. The Shapiro–Wilk test showed that all data, including the changes in the concentrations of individual markers in the case of brain glioma and leukemia, had non-normal distributions. We, therefore, used non-parametric tests for further analyses. 

**Figure 2:**
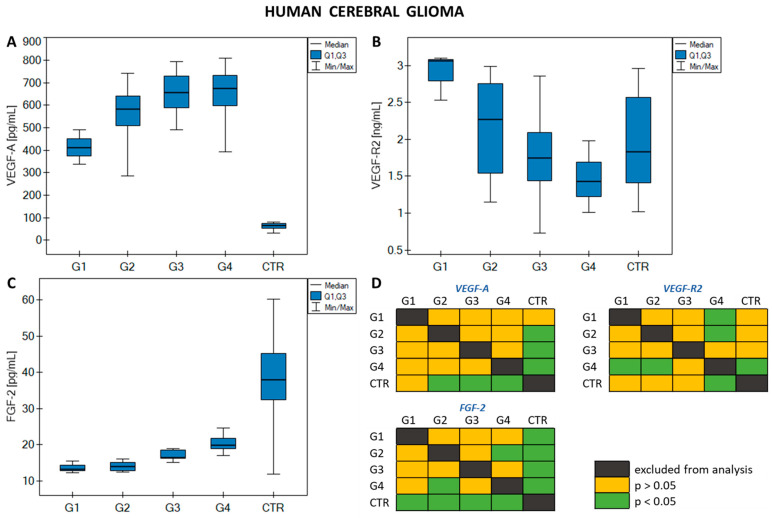
Graphs showing changes in (**A**) VEGF-A concentrations; (**B**) VEGF-R2; (**C**) FGF-2 in human cerebral glioma in relation to disease grade and control group; (**D**) graphical representation of the Dunn–Bonferroni post hoc test.

These were the Kruskal–Wallis test (with an additional post hoc Dunn–Bonferroni test) for the glioma study, and the Mann–Whitney U test for the leukemia study. In the case of brain glioma, high statistical significance (*p* < 0.05) was demonstrated in the overall understanding of the differences between individual glioma grades and between those grades and the control group, while the post hoc test revealed between which data sets these differences were actually statistically significant. These results are presented in Figure 2 and in Table A1.

The above figure shows that statistically significant changes are observed between the control group (CTR) and grades G2–G4 for VEGF-A, between CTR and grade G4, and between grade G4 and grades G1 and G2 for VEGF-R2, and between CTR and all grades of glioma, and between G2 and G4 for FGF-2. In general, VEGF-A concentrations remain constant and similar for grades G2–G4, with a decrease in VEGF-A concentration observed for G1 and CTR. In the case of VEGF-R2, a gradual decrease in concentration was observed from grade G1 to G4, with the CTR concentration comparable to the G2 level. The FGF-2 concentration was similar in stages G1 and G2, increased slightly with increasing grade of glioma malignancy, and was highest in CTR.

**Figure 3:**
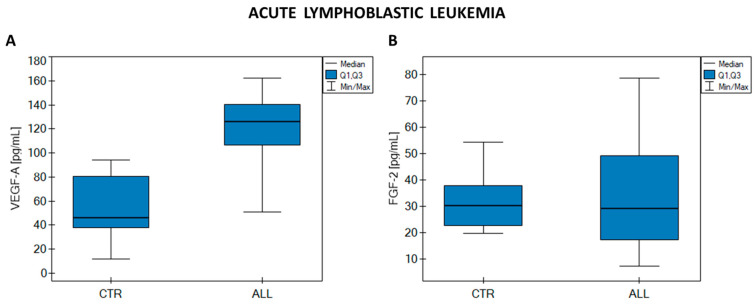
Graphs showing changes in (**A**) VEGF-A concentrations; (**B**) FGF-2 in acute lymphoblastic leukemia (ALL) and the control group (CTR).

Statistical analysis of VEGF-A and FGF-2 concentrations (the Mann–Whitney U test) revealed a statistically significant difference between VEGF-A concentrations in the ALL sample group and the CTR sample group (*p* < 0.01). No such relationship was observed for FGF-2 (*p* = 0.976). The results of the analysis are presented in Figure 3 and in Table A2.

The median VEGF-A concentration is clearly higher in ALL than in CTR, while the median FGF-2 concentrations in the ALL and CTR groups remain at almost the same level. 

A multiple regression model was used to examine the influence of a number of independent variables characterizing a given patient on the concentrations of VEGF-A, VEGF-R2, and FGF-2. These variables were age, Karnofsky score, physical activity, frequency of eating products from plastic packaging, frequency of eating vegetables and fruits, frequency of meat consumption, frequency of smoking tobacco products (in pack-years), and the size (surface area) of the cancer tumor. The aim was to determine the influence of these variables on the concentrations of the analyzed markers, and thus indirectly to characterize the influence of the environment and the patient’s behavior and habits on the overall risk of developing brain glioma. For this reason, data from CTR were not included in the analysis, and the set of data from patients with brain glioma was not divided by disease grade. Detailed results of the statistical analysis are presented in Table A3. The first stage of the analysis was to verify the statistical significance of individual variables in the model. For this purpose, a Student t-test was performed. The test did not show statistical significance for any of the biomarkers measured, and therefore, it cannot be assumed that the variables used in the model have a real impact on the concentrations of VEGF-A, VEGF-R2, and FGF-2. Similarly, testing the statistical significance of all model variables using the analysis of variance test (F test) did not produce any statistically significant results. It was next examined whether the set of all variables had an impact on a single independent variable (biomarker concentration). Although the multiple correlation coefficients R are in the range of 0.60–0.68, which would indicate a high positive correlation, the lack of statistical significance of the test means that it would be incorrect to assume such a relationship. The partial correlation coefficients were intended to determine whether specific independent variables were in some way related to the dependent variable while taking into account the correlation of these variables with the remaining variables in the model. Depending on the variable, both positive and negative partial correlation coefficients were observed, ranging from low to very high correlation. However, due to the lack of statistical significance of the test, the above relationships are not significant from a statistical point of view. 

**Figure 4:**
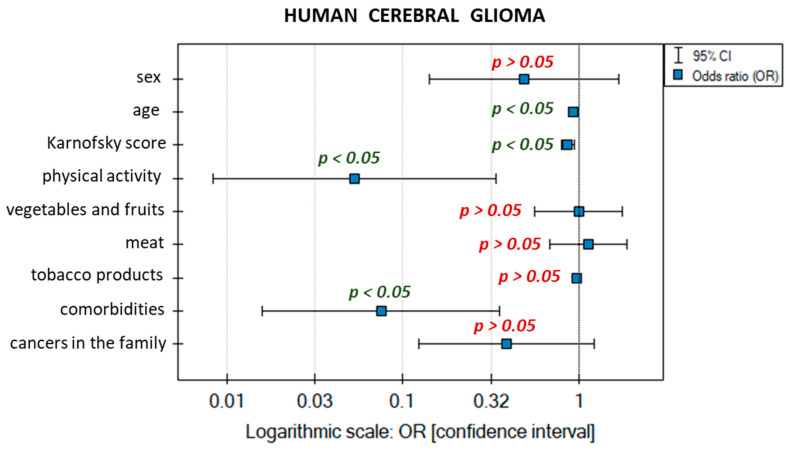
Graphical interpretation of the logistic regression model.

The next stage of the analysis involved building a logistic regression model for brain glioma samples. This study was again used to determine the influence of multiple independent variables on one dependent variable. The independent variables included gender, age, Karnofsky score, physical activity, frequency of eating vegetables and fruit, frequency of meat consumption, frequency of smoking tobacco products (in pack-years), presence of comorbid non-cancer diseases, and history of cancer in the immediate family. The independent variables were not treated as dummy variables (they were not coded with zero–one values). CTR was also taken into account in the analysis. For the logistic regression model, the dependent variable takes only two values: sick or healthy. The variable corresponding to a person suffering from glioma was used as the distinguished value. For each independent variable in the model, a unit odds ratio (OR) was calculated, which expresses the change in the odds of the occurrence of a distinguished value (in our case, sick) when the independent variable increases by 1 unit. For categorical variables (gender, presence of comorbid non-cancer diseases, and history of cancer in the patient’s immediate family) the line at level 1 indicates the value of the odds ratio for the reference category. The reference categories were (i) gender—male; (ii) comorbidities—none; (iii) family history of cancer—no cancer. The impact of individual variables on the risk of brain glioma is presented in the chart showing OR values (Figure 4).

The data describing the logistic regression model obtained are given in Table 3.

The quality of the model fit is moderate (pseudo R^2^, R^2^ Nagelkerke, and R^2^ Cox–Snell values) and very highly statistically significant (likelihood ratio test). The result of the Hosmer–Lemeshow test does not show statistical significance, but at the same time, this is a desirable phenomenon that indicates the similarity of observed numbers and predicted probabilities.

The chance of an increase in the risk of developing brain glioma potentially depends on the analyzed variables characterized by OR values (only statistically significant ones were interpreted). The patient’s age is close to OR = 1, which would mean that the variable has almost no impact on the risk of developing brain glioma, but the result is statistically significant, which suggests that the higher the patient’s age, the lower the chance of developing brain glioma (OR = 0.926). The Karnofsky score is characterized similarly—as the score on a scale from 0 to 100 increases, the likelihood of malignant grades of brain glioma decreases (OR = 0.861, meaning that the odds decrease by a factor of 0.861). Physical activity was measured on an intensity scale: from no physical activity to intense regular exercise. The OR for this variable is OR = 0.053, so as the intensity of regular physical exercise increases, the chance of developing brain glioma decreases by a factor of 0.053. The absence of non-cancer comorbidities has a comparable impact. The OR of 0.075 suggests a 0.075-fold decrease in the chance of developing brain glioma compared with people suffering from comorbidities. The remaining variables have no real impact on the distinguished value: they are statistically insignificant, or their confidence intervals contain the value 1.

Then, mutual correlations (Spearman’s monotonic dependence) between the analyzed proteins were determined, for samples from patients diagnosed with brain glioma and ALL. The study aimed to find potential specific relationships between VEGF-A, VEGF-R2, and FGF-2 in the presence of disease; therefore, to avoid affecting the strength of the correlation, control groups were omitted in the case of both diseases. The correlations are summarized in Table 4.

There was a statistically significant high negative correlation between FGF-2 and VEGF-A and a statistically significant moderate negative correlation between VEGF-A and VEGF-R2 in brain glioma. Negative correlations indicate that an increase in the concentration of one protein will induce a decrease in the concentration of the other. However, there was no correlation between concentrations of FGF-2 and VEGF-R2 in brain glioma or between concentrations of FGF-2 and VEGF-A in ALL.

**Figure A1:**
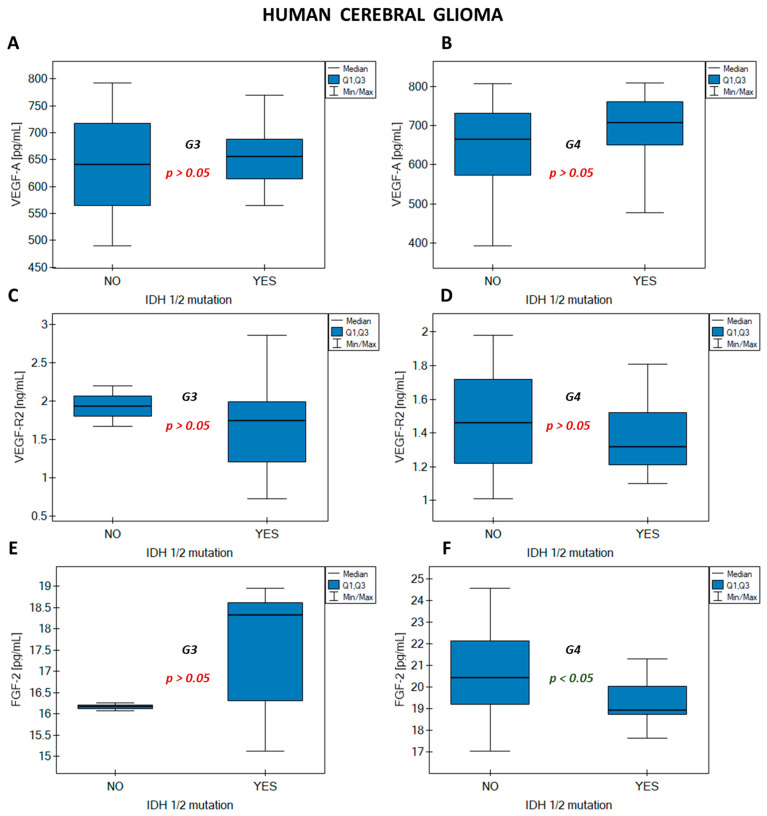
Effect of IDH 1/2 mutation on the concentration of (**A**) VEGF-A grade G3; (**B**) VEGF-A grade G4; (**C**) VEGF-R2 grade G3; (**D**) VEGF-R2 grade G4; (**E**) FGF-2 grade G3; (**F**) FGF-2 grade G4.

The influence of one of the most important molecular markers (IDH 1/2 mutation) on the concentrations of the analyzed protein markers was investigated using the Mann–Whitney U test. The analysis was performed for grades G3 and G4 because in milder grades the IDH 1/2 mutation was absent (G1) or was present in all patients (G2). A statistically significant difference in concentrations between samples from patients with the IDH 1/2 mutation and samples in which the mutation was not recorded was observed only for FGF-2 in grade G4. The presence of the IDH 1/2 mutation in samples resulted in a decrease in FGF-2 concentration. For the remaining proteins (VEGF-A and VEGF-R2) there were no statistically significant differences in concentrations in the presence or absence of the IDH 1/2 mutation. The results are presented in Figure A1 in Appendix A.

**Figure 5:**
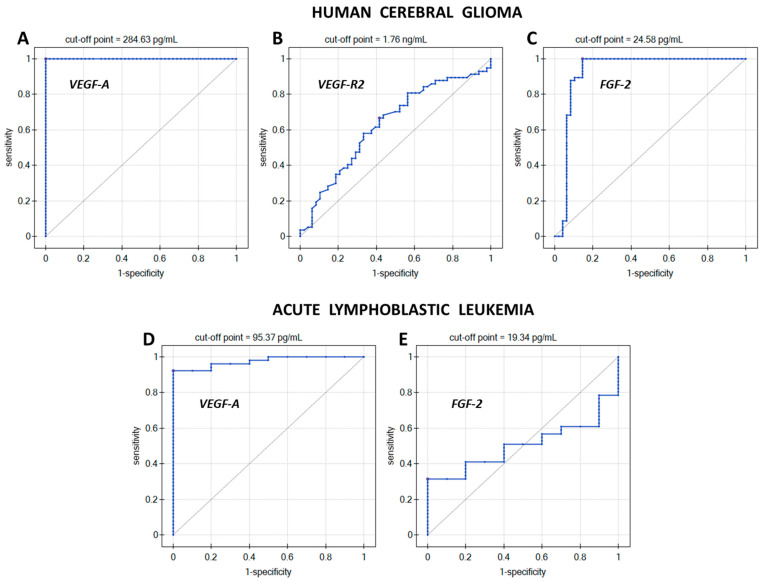
ROC curves for (**A**) VEGF-A in glioma; (**B**) VEGF-R2 in glioma; (**C**) FGF-2 in glioma; (**D**) VEGF-A in ALL; (**E**) FGF-2 in ALL.

The diagnostic usefulness of the methods for determining VEGF-A, VEGF-R2, and FGF-2 in brain glioma and VEGF-A and FGF-2 in ALL was verified by ROC analysis. ROC curves with a marked cut-off point were plotted, as shown in Figure 5. Table 5 presents data on the direction of the diagnostic variable, AUC, sensitivity, specificity, positive predictive value (PPV), negative predictive value (NPV), cut-off point, and *p*-value.

The direction of the diagnostic variable determines whether, as the biomarker concentration increases, the chances of a given phenomenon occurring increase (stimulant) or decrease (destimulant). The direction of the diagnostic variable for VEGF-A concentration in both glioma and ALL is increasing (stimulant). This means that VEGF-A concentration values equal to or above the cut-off point will indicate the presence of the disease. However, the direction of the diagnostic variable for VEGF-R2 and FGF-2 is decreasing (destimulant), and so, in these cases, the disease will be indicated by biomarker concentrations that are less than or equal to the cut-off point. The closer the area under the ROC curve (AUC) is to 1, the more accurately a given sample can be assigned to the group of patients or healthy people, while AUC = 0.50 may suggest a purely random assignment to one of the groups. In Table 4, AUC = 0.50 was observed for FGF-2 determinations in ALL, but due to the lack of statistical significance of the ROC analysis, the contribution of changes in FGF-2 concentrations in ALL was omitted in further considerations. Sensitivity and specificity are very good measures for assessing the accuracy of a given diagnostic test (assay method). VEGF-A and FGF-2 tests provide 100% certainty in detecting people actually suffering from brain glioma, while in the case of ALL, VEGF-A enabled the detection of 92.2% of people with the disease. The maximum 100% certainty of detecting potential healthy people is provided by testing the concentration of VEGF-A (both glioma and ALL). The remaining proteins (VEGF-R2 and FGF-2) allow the detection of people without brain glioma in the sample population at levels of 58.3% and 85.4%, respectively. The positive predictive value (PPV) measures the probability that a person had the disease given a positive test result, while the negative predictive value measures the probability that the disease was not present given a negative test result. Accordingly, 100% probability for PPV and NPV is obtained when measuring VEGF-A in brain glioma. For VEGF-A tests in leukemia, the PPV is 100%, but the NPV is 71.4%. Determinations of the other two proteins in glioma give the probability of the actual occurrence of the disease with a positive test result as 65.5% for VEGF-R2 and 89.1% for FGF-2. However, a person with a negative brain glioma test result based on VEGF-R2 tests can be 59.6% certain that they do not have the disease, and in the case of FGF-2 tests, they have 100% certainty.

## 3. Discussion

The present study has examined the influence of the degree of malignancy of brain glioma and patients’ habits and predisposition to the disease, based on family history of cancer and on the concentrations of the growth factors VEGF-A and FGF-2 and the VEGF-R2 receptor. The influence of the presence of mutations in the IDH 1/2 molecular marker on changes in the concentration of the determined growth factors and receptor was also investigated, as well as the mutual correlations between the analyzed proteins. VEGF-A and FGF-2 concentration levels were also determined in samples from children with ALL, whose values were compared with the control group. Moreover, the relationships between VEGF-A and FGF-2 in the tested ALL samples were characterized.

In the statistical analysis, statistically significant differences were observed between the concentrations of VEGF-A, VEGF-R2, and FGF-2 with respect to the entire sample population and increasing malignancy of brain glioma. The Dunn–Bonferroni post hoc test showed statistically significant differences in concentrations between the grades of glioma and how the concentrations of the proteins compared to the control group, with a clear reduction in VEGF-A concentration in CTR. Significant differences were observed: (i) for VEGF-A between CTR and glioma grades G2 to G4; (ii) for VEGF-R2 between stages G1 and G4 and G2 and G4, as well as between G4 and CTR; and (iii) for FGF-2 between all grades of malignancy and CTR, with a clear increase in concentration values observed for CTR and between G2 and G4. The significantly increased levels of VEGF-A in glioma can be explained by the fact that it plays a key role in the angiogenesis process [32,33]. On the other hand, a decrease in VEGF-R2 concentration with increasing glioma grade was observed, along with a relatively high VEGF-R2 level in CTR. During the growth of a glioma, the process of neoangiogenesis occurs, in which new blood vessels are formed from existing ones to provide the cancer tumor with constant access to nutrients and oxygen. Neoangiogenesis is primarily mediated by cytokines from the VEGF family and their receptors. VEGF-A binds to its main receptor, VEGF-R2 [34]. Therefore, we expect that a significant increase in VEGF-A concentration will cause a decrease in VEGF-R2 concentration, due to the excess of the VEGF cytokine, which has a high affinity for the VEGF-R2 receptor. This hypothesis seems to be confirmed by the result obtained for the correlation between VEGF-A and VEGF-R2, which indicates a moderate negative correlation. Similarly, a high negative correlation was recorded between FGF-2 and VEGF-A concentrations. Both growth factors have a synergistic effect and the concentrations of both increase as the grade of glioma increases. They interact closely in tumor growth and progression [35], and our study also showed that they strongly interact with each other: an increase in the concentration of VEGF-A induced a decrease in the concentration of FGF-2, while a decrease in the concentration of VEGF-A caused an increase in the concentration of FGF-2, as is clearly observed when analyzing the concentrations of these growth factors in the control group for brain glioma.

When comparing the results for VEGF-A and FGF-2 concentrations in ALL and CTR, statistically significant differences were noted only for VEGF-A concentration, the median concentration of which was approximately 2.7 times higher in ALL than in CTR. We, therefore, conclude that VEGF-A has a real and significant impact on the development of this hematological cancer, while FGF-2 appears to have no effect on the mechanisms of progression and development in ALL.

To investigate the influence of the patient’s living environment and habits, an analysis was made of data on the size of the tumor; the patient’s age; Karnofsky score; physical activity; frequency of eating food products from plastic packaging; frequency of eating meat, vegetables, and fruit; and intensity of cigarette smoking. It was concluded that none of the variables has a significant impact on the actual risk of developing brain glioma. However, the logistic regression model indicates further relationships involving an increase or decrease in the chances of developing brain glioma. The model shows that the chances of developing brain glioma decrease with age. The chances of malignant grades of the disease also decrease with the improvement of the general condition of the body and the quality of life as determined by the Karnofsky score. Neither intense physical activity nor a complete lack of such activity contributes to the risk of developing the disease. Similarly, other non-cancer comorbidities and the presence of cancer (other than glioma) in the patient’s immediate family have no influence.

The IDH 1/2 mutation has a statistically significant effect on the FGF-2 concentration in G4 glioma, causing a decrease in the IDH 1/2 concentration. VEGF-A and its receptor VEGF-R2 did not show a relationship similar to that of FGF-2 concentration towards IDH 1/2 mutations, and they can, therefore, be considered independent biomarkers of brain glioma.

The ROC analysis indicated which of the diagnostic tests (assay methods) gives the highest probability of correctly diagnosing brain glioma by measuring only one of the biomarkers. Maximum 100% sensitivity, specificity, PPV, and NPV were obtained only for the determination of VEGF-A in brain glioma, and an increase in the concentration of this biomarker indicates the disease. The remaining diagnostic tests performed less well, and none of them separated the patient group from the control group so effectively. The FGF-2 test in leukemia was completely excluded from the statistical analysis due to the lack of statistical significance of the ROC analysis. For the test for determining VEGF-R2 and FGF-2 in glioma, a decrease in the concentration of the measured protein will indicate the disease because the direction of the diagnostic variable for these tests is decreasing (indicating a destimulant).

## 4. Materials and Methods

### 4.1. Biological Material

A total of 57 plasma samples were used for the glioma experiments. There were 3 grade G1 samples, 10 grade G2 samples, 7 grade G3 samples, and 37 grade G4 samples. The control group (CTR) consisted of 48 plasma samples, which consisted of heavy smokers without diagnosed cancer. The samples came from the Biobank of the Medical University of Bialystok, and consent to carry out the study was obtained from the relevant bioethics committee (approval R-I-002/600/2019). Table A4 presents the clinical characteristics of the patients from whom the research material was taken. 

The study of changes in VEGF-A and FGF-2 concentrations was performed on 51 serum samples of children with acute lymphoblastic leukemia (age 4–10 years) and 10 serum samples from a control group (CTR) (age 4–10 years). The samples came from the Department of Pediatric Oncology and Hematology, Medical University of Bialystok. Consent for the use of this biological material was also received from the bioethics committee (approval APK.002.243.2022).

### 4.2. Procedure for Quantification of VEGF-A, VEGF-R2 and FGF-2

Quantitative analysis of the selected biomarkers was performed using an SPRi analyzer and previously constructed and validated biosensors, sensitive to VEGF-A and FGF-2 [36] and VEGF-R2 [26,37], with linker layers already prepared. Briefly, the first stage of the procedure was the immobilization of ligands on the surface of the biosensors—monoclonal antibodies capturing VEGF-A, FGF-2, or VEGF-R2 from the solution. Ligand solutions prepared according to the recommended procedure were applied to the measurement sites of the biosensors (the volume of each solution is 3 µL) and incubated at 37 °C for an hour. During waiting for the end of the incubation stage, sample preparation began. The sample preparation for each measurement consisted of dilution in PBS buffer so that the detector response signal was within the linear range of the calibration curve for the method used. Therefore, all plasma and serum samples were diluted 2-fold, with the exception of glioma samples for VEGF-A determinations, which were diluted 10-fold. After an hour of incubation, the surface of the active sites was washed once with HBS-ES buffer and once with deionized water. The biosensor prepared in this way was placed on the prism of the device, and an image of the biosensor measurement sites was recorded (data acquisition) at one previously determined SPR angle. Then, 3 µL of the prepared sample was placed on these sites and left for the time specified in the procedure requirements (for VEGF-A, 6 min; for FGF-2, 8 min and 8 min of reaction with the secondary antibody; for VEGF-R2, 10 min). After the time necessary for antibody–analyte interaction had elapsed, the surface of the active sites was washed once with HBS-ES solution and once with deionized water. Then data acquisition began again. The collected data were processed using free ImageJ software (NIH, version 1.8.0_172).

### 4.3. IDH 1/2 Mutation, p53 Gene Mutation and EGFR Expression

Data on mutations or expression of molecular markers were extracted from medical records provided with the samples.

### 4.4. Clinical Data Collection

Clinical data were collected by medical staff of the Biobank of the Medical University of Bialystok. An appropriate questionnaire was prepared, and patients included in the study were asked questions and asked to specify the frequency of a given activity. Therefore, three levels of physical activity have been distinguished—no, moderate, and intense activity; 6 levels of frequency of consumption of vegetables, fruits, and products from plastic packaging—never, less than once a month, once a month, once a week, several times a week, every day; the frequency of meat consumption was determined by the number of days per week from 0 to 7; tobacco product abuse was counted in pack-years smoked; to questions about non-cancer comorbidities and cancer in the immediate family, there were two answers—yes or no, with an indication of the possible disease. The number of points on the Karnofsky scale was determined on the basis of a score from 0 to 100, with the number of points assigned on the basis of the questionnaire, medical records, and patient observation, suggested by the point gradation presented in [38].

### 4.5. Statistical Analysis

Statistical analysis was performed using PQStat software (2023, v.1.8.6.116). Each analysis assumed a level of statistical significance of α = 0.05.

The following statistical tests were performed: (i) Shapiro–Wilk test to examine the normality of the distribution of the analyzed data; (ii) Kruskal–Wallis test checking the hypothesis about the equality of median concentrations of determined cytokines in relation to the division into glioma grades and the control group (H_0_—all medians are equal; H_1_—not all medians are equal); (iii) Dunn–Bonferroni post-hoc test to indicate which glioma grades have significant concentration differences; (iv) Mann–Whitney U test testing the hypothesis about the equality of the median concentrations of the determined cytokines between ALL and CTR and the hypothesis about the equality of the median concentrations of the determined cytokines from plasma samples of glioma patients with and without the IDH 1/2 mutation (H_0_—the median concentrations are equal; H_1_—median concentrations are not equal); (v) multiple regression model to examine the influence of independent variables characterizing the patient (data from the clinical description of samples) on the concentrations of markers, which returns the value of the multiple correlation coefficient R, then interpreted according to the J. Guilford scale; (vi) a logistic regression model examining the impact of the patient’s living environment and habits on the risk of developing glioma, which is determined by the unit odds ratio OR; (vii) Spearman’s rank correlation to examine the strength of interactions between individual proteins, defined as Spearman’s correlation coefficient *r*, interpreted according to J. Guilford scale; (viii) ROC analysis to check the diagnostic usefulness of the methods for determining the tested cytokines, which is characterized by AUC, PPV, NPV, sensitivity and specificity.

## 5. Conclusions

Our team confirmed the existence of significant differences in the concentrations of pro-angiogenic cytokines, such as VEGF-A, VEGF-R2, and FGF-2, in brain glioma in relation to the stage of the disease and the control group. These differences are especially visible between milder stages of glioma (G1–G2) and aggressive grades, where the disease is already very advanced (G3–G4), and between the control group and advanced grades. In the case of ALL, only VEGF-A concentrations change significantly, where the median concentration in the patient group is approximately 2.7 times higher than in the control group. The determined cytokines interact moderately or highly, showing negative correlations, so an increase in the concentration of one of them causes a decrease in the concentration of the other one. Neither diet nor the intensity of physical activity increase the risk of developing brain glioma. There is also no impact of the presence of other non-cancer diseases in the patient or cancers other than glioma in his immediate family. However, the logistic regression model shows that the risk of developing glioma is lowest in elderly people, and as the Karnofsky scale score increases, the incidence of malignant grades of glioma decreases. The lack of effect of the IDH 1/2 mutation on the concentrations of VEGF-A and VEGF-R2 suggests that both proteins are independent biomarkers, and moreover, the conducted research shows that only the method for determining VEGF-A in glioma is 100% sensitive and specific.

The presented methods for determining the tested cytokines (VEGF-A, VEGF-R2, and FGF-2) could be used primarily to differentiate benign grades of brain glioma from malignant grades, while the VEGF-A method could be used for the diagnosis of ALL. The inferred correlations between proteins provide new knowledge on the interactions of the studied proangiogenic factors in glioma and ALL. These relationships can be taken into account when designing new therapeutic approaches, treatment methods, and the design of subsequent drugs aimed at weakening the effects caused by signaling associated with pro-angiogenic factors.

Currently, there is no ideal diagnostic method that would enable a clear diagnosis to be made. Similarly, the methods proposed by our team have certain limitations. First of all, they do not enable early diagnosis of glioma because the concentration values of the control group and patients in grades G1 and G2 are almost identical. Samples require preliminary preparation, which involves dilution according to the selected method. Nevertheless, our methods enable the determination of up to 12 samples simultaneously with low sample consumption (3 μL of the sample). Biosensors are fully regenerable, so the cost of a single determination is also lower, they use small amounts of reagents and do not require labeling of the analytes being determined.

## Figures and Tables

**Table 1:** Families of gliomas, neuroglial tumors, and neuronal tumors according to WHO CNSH classification.

WHO CNS5 Classification	Short Characteristic	Reference
adult-type diffuse gliomas	constitute the majority of primary neuro-oncological tumors in adults, an example of which is glioma, wild-type IDH	[9]
diffuse low-grade gliomas of the pediatric type	have a good prognosis	
diffuse high-grade pediatric gliomas	tumors of an aggressive nature	
limited astrocytic gliomas	characterized by a more constant growth pattern, unlike diffuse gliomas	
glioneuronal and neuronal tumors	a diverse group of tumors	
ependymomas	classified based on the site of occurrence and histological and molecular features	

**Table 2:** Classification of glioma grades according to WHO CNS5.

Histological Type of Tumor	Tumor Type	Grade
Subependymomas Pilocytic astrocytoma	Astrocytoma	CNS WHO grade 1
Oligodendroglioma	Oligodendroglioma, IDH-mutant and 1p/19q-codeleted	CNS WHO grade 2
Diffuse astrocytoma	Astrocytoma, IDH-mutant	
Anaplastic oligodendroglioma	Oligodendroglioma, IDH-mutant and 1p/19q-codeleted	CNS WHO grade 3
Anaplastic astrocytoma	Astrocytoma, IDH-mutant	
Astrocytoma	Astrocytoma, IDH-mutant and CDKN2A/B homozygous deletion	CNS WHO grade 4
Astrocytoma	Glioblastoma, IDH-wildtype and TERT promoter mutation, EGFR amplification, or gain/loss of chromosome 7/10	
Glioblastoma	Glioblastoma, IDH-wildtype	

**Table 3:** Data characterizing the logistic regression model.

Human Cerebral Glioma		
Parameter	Value	
Likelihood ratio test	*p* < 0.01	
Model fit quality	pseudo R^2^ = 0.43	
	R^2^ (Nagelkerke) = 0.60	
	R^2^ (Cox-Snell) = 0.45	
Hosmer-Lemeshow test	*p* = 0.93	
Independent Variable	*p*-Value	Odds Ratio (OR)
age	*p* < 0.05	0.926
Karnofsky score		0.861
physical activity		0.053
comorbidities		0.075

**Table 4:** Correlations between determined proteins. Interpretation of *r* coefficients based on J. Guilford’s scale.

Relationship between Proteins	Correlation Strength [*r*]	*p*-Value
**Human Cerebral Glioma**		
FGF-2—VEGF-A	−0.61 high negative correlation	<0.01
FGF-2—VEGF-R2	0.04 no correlation	>0.05
VEGF-A—VEGF-R2	−0.30 average negative correlation	<0.01
**Acute Lymphoblastic Leukemia**		
FGF-2—VEGF-A	−0.1 weak negative correlation	>0.05

**Table 5:** Data characterizing the ROC analysis. AUC—Area Under Curve; PPV—Positive Predictive Value; NPV—Negative Predictive Value.

Human Cerebral Glioma								
Determination Method	Direction of the Diagnostic Variable	AUC	Sensitivity [%]	Specificity [%]	PPV [%]	NPV [%]	Cut-Off Point	*p*-Value
VEGF-A	stimulant	1.00	100	100	100	100	284.63 pg/mL	<0.01
VEGF-R2	destimulant	0.63	66.7	58.3	65.5	59.6	1.76 ng/mL	<0.05
FGF-2	destimulant	0.93	100	85.4	89.1	100	24.58 pg/mL	<0.01
**Acute Lymphoblastic Leukemia**								
VEGF-A	stimulant	0.97	92.2	100	100	71.4	95.37 pg/mL	<0.01
FGF-2	destimulant	0.50	31.4	100	100	22.2	19.34 pg/mL	>0.05

## Data Availability

Data are contained within the article.

## Appendix A

**Table A1:** Details of the Kruskal-Wallis test for Figure 2. Q1—lower quartile; Q3—upper quartile; Min—minimum concentration value; Max—maximum concentration value; IQR—interquartile range.

Human Cerebral Glioma					
**VEGF-A**					
Glioma grade or CTR	G1	G2	G3	G4	CTR
Median (Q2)	412.46	583.59	655.44	675.48	64.99
Q1	374.94	508.84	589.50	598.45	52.48
Q3	451.64	640.14	728.87	733.21	74.58
Min	337.42	284.63	489.87	392.18	31.32
Max	490.81	743.03	792.23	809.03	81.40
IQR	76.70	131.30	139.37	134.76	22.10
**VEGF-R2**					
Glioma grade or CTR	G1	G2	G3	G4	CTR
Median (Q2)	3.06	2.27	1.75	1.43	1.84
Q1	2.80	1.55	1.44	1.22	1.41
Q3	3.08	2.76	2.10	1.69	2.57
Min	2.53	1.15	0.73	1.01	1.02
Max	3.10	2.99	2.86	1.98	2.96
IQR	0.28	1.21	0.66	0.47	1.16
**FGF-2**					
Glioma grade or CTR	G1	G2	G3	G4	CTR
Median (Q2)	13.23	14.00	16.31	19.92	37.90
Q1	12.68	12.70	16.17	18.79	32.37
Q3	14.32	15.13	18.47	21.75	45.20
Min	12.13	12.34	15.12	17.02	11.82
Max	15.40	16.07	18.95	24.58	60.24
IQR	1.64	2.43	2.30	2.96	12.83

**Table A2:** Details of the Mann-Whitney U test for Figure 3. Q1—lower quartile; Q3—upper quartile; Min—minimum concentration value; Max—maximum concentration value; IQR—interquartile range.

Acute Lymphoblastic Leukemia		
**VEGF-A**		
ALL or CTR	ALL	CTR
Median (Q2)	125.89	46.11
Q1	106.31	37.63
Q3	140.26	80.73
Min	50.93	11.70
Max	162.36	94.12
IQR	33.95	43.10
**FGF-2**		
ALL or CTR	ALL	CTR
Median (Q2)	29.19	30.25
Q1	17.40	22.83
Q3	49.28	37.89
Min	7.35	19.87
Max	78.56	54.22
IQR	31.88	15.06

**Table A3:** Characteristics of the multiple regression model.

Human Cerebral Glioma						
**VEGF-A**						
Independent Variables	R	F	*p*	t-Student	Partial Correlation	*p*
age	0.60	1.57	>0.05	0.38	0.08	>0.05
Karnofsky score				−2.25	−0.43	
physical activity				−1.48	−0.30	
frequency of consuming products with plastic packaging				−0.52	−0.11	
frequency of eating vegetables and fruits				0.80	0.17	
frequency of meat consumption				−0.37	−0.79	
frequency of smoking tobacco products				0.71	0.15	
size of the cancer tumor				0.61	0.13	
**VEGF-R2**						
age	0.63	1.81	>0.05	−1.17	−0.24	>0.05
Karnofsky score				1.37	0.28	
physical activity				−0.90	−0.18	
frequency of consuming products with plastic packaging				−1.04	−0.21	
frequency of eating vegetables and fruits				−0.30	−0.06	
frequency of meat consumption				1.07	0.22	
frequency of smoking tobacco products				0.05	0.01	
size of the cancer tumor				0.72	0.15	
**FGF-2**						
age	0.68	2.34	>0.05	1.41	0.29	>0.05
Karnofsky score				−1.45	−0.30	
physical activity				−0.98	−0.20	
frequency of consuming products with plastic packaging				0.63	0.13	
frequency of eating vegetables and fruits				−1.22	−0.25	
frequency of meat consumption				−1.70	−0.34	
frequency of smoking tobacco products				0.94	0.20	
size of the cancer tumor				0.61	0.13	

**Table A4:** Clinical characteristics of patients from whom glioma samples and control samples were obtained.

Human Cerebral Glioma							
Control Group/Tumor Grade							
Variable		K *n* = 48	G1 *n* = 3	G2 *n* = 10	G3 *n* = 7	G4 *n* = 37	Age Difference between K and G1–G4 ^A^
Age (years)	range	39–66	29–43	30–57	38–72	33–77	No statistically significant difference (*p* > 0.05)
	median	61	39	43.5	45	60	
Gender	male	27	1	5	3	26	
	female	21	2	5	4	11	
Tumor size (cm^2^)	<15		2	2	3	10	
	>15		1	2	1	10	
The presence of other neo-plasms in the immediate family	yes		0	6	3	17	
	no		3	4	4	20	
Concomitant non-cancerous diseases	yes		2	3	3	21	
	no		1	7	4	16	
Histological type of tumors	oligodendroglial		0	1	3	0	
	astrocytic		3	9	4	37	
Number of smokers	number of cases	48	1	6	4	22	Pack-year difference between K and G1–G4 ^B^
	pack-years (median)	37.5	0 ^C^	8	26	38.5	No statistically significant difference (*p* > 0.05)
IDH 1/2 mutation	yes		0	10	5	10	
	no		3	0	2	27	
p53 mutation	yes		1	10	7	34	
	no		2	0	0	3	
EGFR expression	yes		0	6	2	29	
	no		3	4	5	8	

^A^ Mann–Whitney U test; ^B^ Mann–Whitney U test; ^C^ one case was 21 pack-years.
